# Thresholds of various glycemic measures for diagnosing diabetes based on prevalence of retinopathy in community-dwelling Japanese subjects: the Hisayama Study

**DOI:** 10.1186/1475-2840-13-45

**Published:** 2014-02-17

**Authors:** Naoko Mukai, Miho Yasuda, Toshiharu Ninomiya, Jun Hata, Yoichiro Hirakawa, Fumie Ikeda, Masayo Fukuhara, Taeko Hotta, Masafumi Koga, Udai Nakamura, Dongchon Kang, Takanari Kitazono, Yutaka Kiyohara

**Affiliations:** 1 Department of Environmental Medicine, Graduate School of Medical Sciences, Kyushu University, 3-1-1 Maidashi, Higashi-ku, Fukuoka 812-8582, Japan; 2 Department of Medicine and Clinical Science, Graduate School of Medical Sciences, Kyushu University, Fukuoka, Japan; 3 Department of Ophthalmology, Graduate School of Medical Sciences, Kyushu University, Fukuoka, Japan; 4 Department of Clinical Chemistry and Laboratory Medicine, Kyushu University Hospital, Fukuoka, Japan; 5 Department of Internal Medicine, Kawanishi City Hospital, Hyogo, Japan

**Keywords:** Diagnostic criteria, Hemoglobin A_1c_, Glycated albumin, 1,5-anhydroglucitol, Fasting plasma glucose, 2-hour postload glucose, Retinopathy

## Abstract

**Background:**

There has been controversy over the diagnostic thresholds of hemoglobin A_1c_ (HbA_1c_) for diabetes. In addition, no study has examined the thresholds of glycated albumin (GA) and 1,5-anhydroglucitol (1,5-AG) for diagnosing diabetes using the presence of diabetic retinopathy (DR). We examined the optimal thresholds of various glycemic measures for diagnosing diabetes based on the prevalence of DR in community-dwelling Japanese subjects.

**Methods:**

A total of 2,681 subjects aged 40-79 years underwent a 75-g oral glucose tolerance test, measurement of HbA_1c_, GA, and 1,5-AG, and an ophthalmic examination in 2007-2008. The associations of glycemic measures with DR status were examined cross-sectionally. DR was assessed by an examination of the fundus photograph of each eye and graded according to the International Clinical Diabetic Retinopathy Disease Severity Scale. We divided the values of glycemic measures into ten groups on the basis of deciles. The receiver operating characteristic (ROC) curve analysis was performed to determine the optimal threshold of each glycemic measure for detecting the presence of DR.

**Results:**

Of the subjects, 52 had DR. The prevalence of DR increased steeply above the ninth decile for fasting plasma glucose (FPG) (6.2-6.8 mmol/l), for 2-hour postload glucose (PG) (9.2-12.4 mmol/l), for HbA_1c_ (5.9-6.2% [41-44 mmol/mol]), and for GA (16.2-17.5%), and below the second decile for 1,5-AG (9.6-13.5 μg/mL). The ROC curve analysis showed that the optimal thresholds for DR were 6.5 mmol/l for FPG, 11.5 mmol/l for 2-hour PG, 6.1% (43 mmol/mol) for HbA_1c_, 17.0% for GA, and 12.1 μg/mL for 1,5-AG. The area under the ROC curve (AUC) for 2-hour PG (0.947) was significantly larger than that for FPG (0.908), GA (0.906), and 1,5-AG (0.881), and was marginally significantly higher than that for HbA_1c_ (0.919). The AUCs for FPG, HbA_1c_, GA, and 1,5-AG were not significantly different.

**Conclusions:**

Our findings suggest that the FPG and HbA_1c_ thresholds for diagnosing diabetes in the Japanese population are lower than the current diagnostic criterion, while the 2-hour PG threshold is comparable with the diagnostic criterion. 2-hour PG had the highest discriminative ability, whereas FPG, HbA_1c_, GA, and 1,5-AG were similar in their ability.

## Background

The International Expert Committee [1,2], the American Diabetes Association [3], and the World Health Organization [4] recently proposed the use of hemoglobin A_1c_ (HbA_1c_) to diagnose diabetes at a threshold of 6.5% (48 mmol/mol). This threshold was based primarily on the findings of several epidemiological studies in Western populations that investigated HbA_1c_ levels associated with a higher prevalence of diabetic retinopathy (DR), the most specific microvascular complication of diabetes [5-7]. It has been reported that a higher HbA_1c_ level was significantly associated with DR in subjects with diabetes [8], and some clinical trials have demonstrated that lowering HbA_1c_ levels decreased the risk of microvascular complications, such as DR, in diabetes patients [9-11]. These findings suggest that HbA_1c_ levels are intimately related to the risk of DR, and this evidence supports the use of HbA_1c_ as a diagnostic tool for diabetes. However, there has been controversy over the diagnostic threshold of HbA_1c_. An integrated study of three general populations has shown that the relation between fasting plasma glucose (FPG) levels and the prevalence of retinopathy was continuous, with no clear threshold [12], whereas a prospective study of a French population recently revealed that the optimal threshold of HbA_1c_ for incident retinopathy was 6.0%, which is below the current diagnostic criterion [13]. In addition, several cross-sectional studies of Asian populations, including our previous study, have examined this issue [14-18], but the optimal HbA_1c_ thresholds have differed among these investigations. Thus, a reevaluation of threshold of HbA_1c_ for DR is needed.

Glycated albumin (GA) and 1,5-anhydroglucitol (1,5-AG) levels, which are serum markers of hyperglycemia, have also been found to be significantly associated with microvascular complications [19,20]. There have been a few studies investigating GA [21-23] and 1,5-AG levels [24-26] to detect subjects with glucose intolerance defined by glucose levels, but no study has examined the diagnostic thresholds of these glycemic measures for diabetes based on the presence of DR, and it is uncertain whether GA and 1,5-AG measurements are applicable as a diagnostic tool for diabetes [27,28]. In addition, in the general Asian community, there are limited data assessing FPG and 2-hour postload glucose (PG) levels associated with the prevalence of DR [14,17,29].

The purposes of this study were to determine the thresholds of FPG, 2-hour PG, HbA_1c_, GA, and 1,5-AG for the diagnosis of diabetes based on the prevalence of DR in a community-dwelling Japanese population, and to compare the ability of these five glycemic measures to differentiate subjects with and without DR.

## Methods

### Study population

A population-based prospective study of cardiovascular disease and its risk factors has been underway since 1961 in the town of Hisayama, a suburb of the Fukuoka metropolitan area on Japan’s Kyushu Island. The population of the town has been stable for 50 years and was approximately 8,400 in 2010. The age and occupational distributions, and nutritional intake of the population were similar to those of Japan as a whole based on data from the national census and nutrition survey [30,31]. In 2007 and 2008, a cross-sectional survey for the present study was performed in the town. A detailed description of this survey was published previously [30]. There were a total of 3,835 residents aged 40-79 years based on the town registry, and 2,957 (participation rate, 77.1%) took part in a comprehensive assessment, including a 75-g oral glucose tolerance test (OGTT), the measurement of HbA_1c_, and an ophthalmic examination. We excluded the eight subjects who did not consent to participate in the study, 46 who had already had breakfast, 35 who were on insulin therapy, and 156 who refused the OGTT, leaving a total of 2,712 subjects who completed both the 75-g OGTT and HbA_1c_ measurement. Among these, 21 subjects who lacked ophthalmic examination information and 10 for whom there was no measurement of GA or 1,5-AG were excluded, and the remaining 2,681 subjects (1,192 men, 1,489 women) were enrolled in the present study.

### Clinical evaluation and laboratory measurements

The study subjects underwent the OGTT between 8:00 and 10:30 A.M. after an overnight fast of at least 12 hours. Blood for the glucose assay was obtained by venipuncture into tubes containing sodium fluoride at fasting and at 2-hour postload, and was separated into plasma and blood cells within 20 min. Plasma glucose concentrations were determined by the hexokinase method. According to the 1998 World Health Organization criteria [32], diabetes was defined as FPG ≥7.0 mmol/l or 2-hour PG ≥11.1 mmol/l or both, or the use of antidiabetic medications. Those who were diagnosed with diabetes with or without treatment before the examination were considered to be cases of known diabetes. Blood samples were also collected for the determination of HbA_1c_ levels, hemoglobin (Hb) and serum creatinine concentrations. HbA_1c_ levels were measured by latex aggregation immunoassay (Determiner HbA1C; Kyowa Medex, Tokyo, Japan). The values for HbA_1c_ were estimated as a National Glycohemoglobin Standardization Program (NGSP) equivalent value calculated with the formula: HbA_1c_ (%) = 1.02 × HbA_1c_ (Japan Diabetes Society [JDS]) (%) + 0.25% [33]. A portion of each serum specimen was stored at -80°C for 5 years until it was used for measurement of GA and 1,5-AG in 2012. Serum GA levels were determined by an enzymatic method using an albumin-specific proteinase, ketoamine oxidase, and an albumin assay reagent (Lucica GA-L; Asahi Kasei Pharma, Tokyo, Japan). Serum 1,5-AG concentrations were measured enzymatically (Lana 1,5AG Auto Liquid; Nippon Kayaku, Tokyo, Japan). Hb concentrations were measured by sodium lauryl sulfate-hemoglobin method. Anemia was defined as Hb <13.0 g/dL for men and <12.0 g/dL for women [34]. Serum creatinine concentrations were measured enzymatically, and estimated glomerular filtration rate (eGFR) was calculated using the following new Japanese equation: eGFR (mL/min/1.73 m^2^) = 194 × (serum creatinine [mg/dL])^−1.094^ × (age [years])^−0.287^ × (0.739 if female) [35]. Renal failure was defined as an eGFR <30 mL/min/1.73 m^2^ (chronic kidney disease stage 4 or 5) [36].

The height and weight were measured with the subject in light clothes without shoes, and the body mass index (BMI) (kg/m^2^) was calculated. Each participant completed a self-administered questionnaire covering medical history and antidiabetic treatment.

### Ophthalmic examination and definition of diabetic retinopathy

The methods used for the ophthalmic examination have been described in detail previously [14]. Briefly, each participant underwent fundus photographs for the assessment of DR. After pupil dilatation with 1.0% tropicamide and 10% phenylephrine, fundus photographs (45°) were taken from both eyes of each participant using a Topcon digital TRC NW-6SF fundus camera (Topcon Corporation, Tokyo, Japan). The photographs were taken in 1-field per eye, centered on the macula. The photographs were assessed by photographic graders who were masked to the clinical information. The severity of DR was classified into 5 categories according to the International Clinical Diabetic Retinopathy Disease Severity Scale: no retinopathy (equivalent to the Early Treatment of Diabetic Retinopathy Study [ETDRS] scale level 10), mild nonproliferative DR (equivalent to ETDRS level 20), moderate nonproliferative DR (equivalent to ETDRS levels 35, 43, and 47), severe nonproliferative DR (equivalent to ETDRS levels 53A-53E), and proliferative DR (equivalent to ETDRS levels 61 or higher) [37]. The degree of DR was determined according to the grading in the worse eye. The presence of DR was defined as the presence of mild or moderate or severe nonproliferative DR, or proliferative DR in either eye.

### Statistical analysis

The SAS software package version 9.3 (SAS Institute, Cary, NC) was used to perform all statistical analyses. We assessed the statistical significance of differences in the prevalence or mean of each factor among the DR status groups by using a logistic or linear regression model, respectively. To analyze FPG, 2-hour PG, HbA_1c_, GA, and 1,5-AG levels as categorical variables, these values were divided into ten groups on the basis of deciles. The receiver operating characteristic (ROC) curve analysis was performed to determine the optimal threshold of each glycemic measure for detecting the presence of DR. The optimal threshold was obtained from the point on the ROC curve closest to the ideal of 100% sensitivity and 100% specificity. The discrimination of each measure of glycemia for DR was assessed by the area under the ROC curve (AUC). The difference in the AUC was estimated using the method of DeLong et al. [38]. A value of p < 0.05 was considered statistically significant in all analyses.

### Ethical considerations

This study was conducted with the approval of the Kyushu University Institutional Review Board for Clinical Research, and written informed consent was obtained from all the participants.

## Results

Of the study participants, 52 (1.9%) had DR. Mild nonproliferative DR, moderate nonproliferative DR, severe nonproliferative DR, and proliferative DR were found in 33 (1.2%), 6 (0.2%), 13 (0.5%), and 0 (0%) subjects, respectively. The clinical characteristics of subjects are shown in Table 1. The mean age of participants was 60 years, and men comprised 44.5% of the group. The prevalence of diabetes, known diabetes, anemia, and renal failure was 15.2%, 10.0%, 13.2%, and 0.3%, respectively. The mean values of age, FPG, 2-hour PG, HbA_1c_, GA, diabetes duration and BMI, and the frequencies of men, diabetes, and known diabetes were significantly higher in the subjects with DR than in those without DR, and the subjects with DR had significantly lower 1,5-AG values. The mean values of Hb and eGFR and the frequencies of anemia and renal failure did not differ between the groups.

**Table 1:** Clinical characteristics of subjects, 2007-2008

**Variable**	**Total**	**No retinopathy**	**Diabetic retinopathy**	**p value**
	**(n = 2,681)**	**(n = 2,629)**	**(n = 52)**	
Age (years)	60 (10)	60 (10)	67 (9)	<0.001
Men (%)	44.5	43.9	75.0	<0.001
Fasting plasma glucose (mmol/l)	5.8 (1.1)	5.7 (1.0)	8.7 (2.5)	<0.001
2-hour postload glucose (mmol/l)	7.9 (3.7)	7.7 (3.4)	18.0 (5.3)	<0.001
Hemoglobin A_1c_ (%)	5.5 (0.7)	5.5 (0.7)	7.4 (1.4)	<0.001
(mmol/mol)	37 (8)	36 (7)	57 (15)	<0.001
Glycated albumin (%)	15.2 (2.8)	15.1 (2.4)	22.7 (6.1)	<0.001
1,5-anhydroglucitol (μg/mL)	20.2 (8.3)	20.5 (8.1)	7.7 (7.1)	<0.001
Diabetic retinopathy (%)	1.9	0	100	>0.99
Diabetes (%)	15.2	13.6	96.2	<0.001
Known diabetes (%)	10.0	8.3	94.2	<0.001
Diabetes duration (years)	9.5 (7.9)	8.3 (7.2)	14.8 (9.0)	<0.001
Body mass index (kg/m^2^)	23.2 (3.4)	23.2 (3.4)	24.7 (3.6)	0.002
Hemoglobin (g/dL)	13.6 (1.4)	13.6 (1.4)	13.9 (1.5)	0.09
Anemia (%)	13.2	13.1	17.3	0.37
eGFR (mL/min/1.73 m^2^)	72.9 (13.9)	72.9 (13.8)	71.4 (18.6)	0.44
Renal failure (%)	0.3	0.3	0	0.99

eGFR: estimated glomerular filtration rate. All values are given as the mean (standard deviations) or as a percentage.

Diabetes was defined as fasting plasma glucose of ≥7.0 mmol/l, and/or 2-hour postload glucose of ≥11.1 mmol/l, and/or the use of antidiabetic medication.

Known diabetes was defined as those who were diagnosed with diabetes with or without treatment before the examination.

Anemia was defined as hemoglobin <13.0 g/dL for men and <12.0 g/dL for women.

Renal failure was defined as an eGFR <30 mL/min/1.73 m^2^.

**Figure 1:**
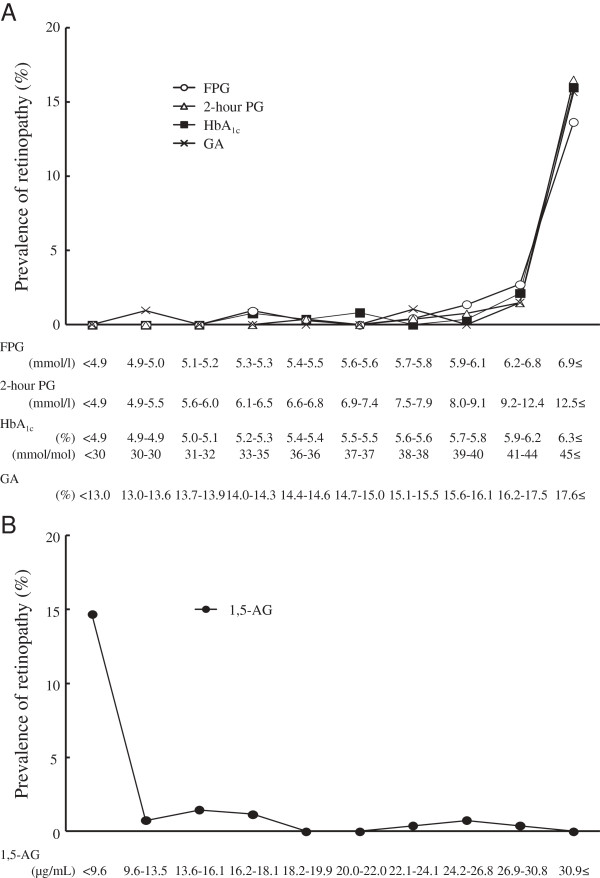
**Prevalence of diabetic retinopathy by deciles of the distribution of fasting plasma glucose, 2-hour postload glucose, hemoglobin A**_**1c**_**, glycated albumin (A), and 1,5-anhydroglucitol levels (B).** FPG: fasting plasma glucose; 2-hour PG: 2-hour postload glucose; HbA_1c_: hemoglobin A_1c_; GA: glycated albumin; 1,5-AG: 1,5-anhydroglucitol.

Figure 1A shows the prevalence of DR by deciles of the distribution of FPG, 2-hour PG, HbA_1c_, and GA levels. The prevalence of DR was very low in the first through eighth deciles for each glycemic measure, but started to increase steeply from the ninth decile for FPG (6.2-6.8 mmol/l), 2-hour PG (9.2-12.4 mmol/l), HbA_1c_ (5.9-6.2% [41-44 mmol/mol]), and GA (16.2-17.5%). Figure 1B demonstrates the prevalence of DR by deciles of 1,5-AG levels. The prevalence of DR increased markedly below the second decile for 1,5-AG (9.6-13.5 μg/mL), while there was no apparent increase in the prevalence of DR between the third and the tenth deciles of 1,5-AG.

The optimal thresholds of each glycemic measure for detecting prevalent DR using ROC curve analyses are shown in Table 2. The optimal threshold was 6.5 mmol/l for FPG, 11.5 mmol/l for 2-hour PG, 6.1% (43 mmol/mol) for HbA_1c_, 17.0% for GA, and 12.1 μg/mL for 1,5-AG. The sensitivity, specificity, positive predictive value, and negative predictive value of these thresholds were 82.7%, 86.6%, 10.9%, and 99.6% for FPG, 90.4%, 89.3%, 14.3%, and 99.8% for 2-hour PG, 86.5%, 88.8%, 13.3%, and 99.7% for HbA_1c_, 86.5%, 89.0%, 13.5%, and 99.7% for GA, and 78.8%, 85.8%, 9.9%, and 99.5% for 1,5-AG_,_ respectively. Among the five glycemic measures, 2-hour PG threshold of 11.5 mmol/l had the highest sensitivity, while 1,5-AG threshold of 12.1 μg/mL showed the lowest sensitivity. In addition, with the exception of the thresholds for 2-hour PG and 1,5-AG, the thresholds of the glycemic measures were not substantially changed when DR was defined as a moderate or higher level of retinopathy (6.5 mmol/l for FPG, 13.1 mmol/l for 2-hour PG, 6.3% [45 mmol/mol] for HbA_1c_, 17.2% for GA, and 10.8 μg/mL for 1,5-AG).

**Table 2:** **Thresholds of FPG, 2-hour PG, HbA**_**1c**_**, GA, and 1,5-AG levels for detecting diabetic retinopathy**

	**Threshold**	**Sensitivity**	**Specificity**	**PPV**	**NPV**
		**(%)**	**(%)**	**(%)**	**(%)**
FPG	6.5 mmol/l	82.7	86.6	10.9	99.6
2-hour PG	11.5 mmol/l	90.4	89.3	14.3	99.8
HbA_1c_	6.1% (43 mmol/mol)	86.5	88.8	13.3	99.7
GA	17.0%	86.5	89.0	13.5	99.7
1,5-AG	12.1 μg/mL	78.8	85.8	9.9	99.5

FPG: fasting plasma glucose; 2-hour PG: 2-hour postload glucose; HbA_1c_: hemoglobin A_1c_; GA: glycated albumin; 1,5-AG: 1,5-anhydroglucitol; PPV: positive predictive value; NPV: negative predictive value.

The optimal threshold was defined as the point on the receiver operating characteristic curve closest to the ideal of 100% sensitivity and 100% specificity.

**Figure 2:**
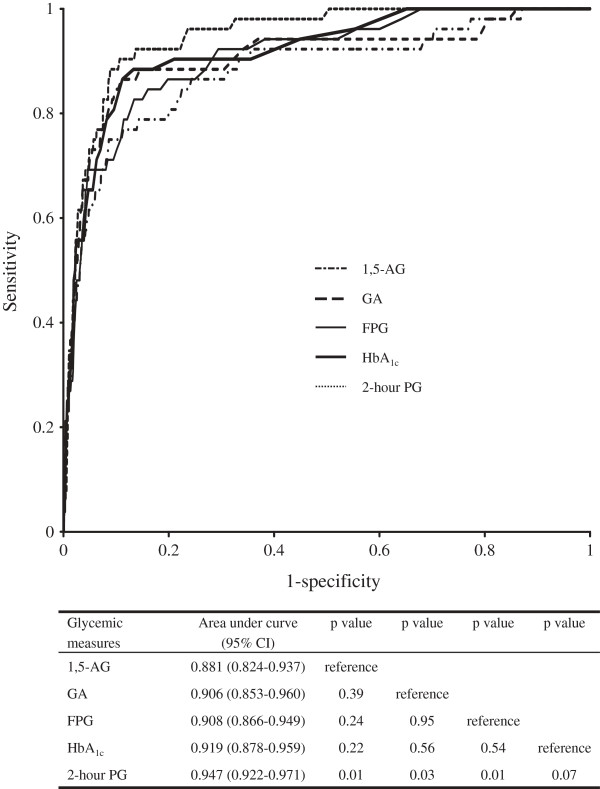
**Comparison of the discriminative ability of fasting plasma glucose, 2-hour postload glucose, hemoglobin A**_**1c**_**, glycated albumin, and 1,5-anhydroglucitol for detecting the presence of diabetic retinopathy.** FPG: fasting plasma glucose; 2-hour PG: 2-hour postload glucose; HbA_1c_: hemoglobin A_1c_; GA: glycated albumin; 1,5-AG: 1,5-anhydroglucitol; CI: confidence interval.

To evaluate the ability of each glycemic measure to identify the presence of DR, we compared the AUC among glycemic measures (Figure 2). The AUC for 2-hour PG was 0.947 (95% confidence interval [CI] 0.922-0.971), which was significantly larger than that for FPG (0.908 [95% CI 0.866-0.949]; p = 0.01), GA (0.906 [95% CI 0.853-0.960]; p = 0.03), and 1,5-AG (0.881 [95% CI 0.824-0.937]; p = 0.01), and was marginally significantly higher than that for HbA_1c_ (0.919 [95% CI 0.878-0.959]; p = 0.07). The AUC for 1,5-AG was lower than that for other measures of glycemia, but there was no significant difference in the AUC among FPG, HbA_1c_, GA, and 1,5-AG.

## Discussion

Using data from a cross-sectional survey in a Japanese community, we demonstrated that the optimal thresholds for detecting prevalent DR from ROC analyses were 6.5 mmol/l for FPG, 11.5 mmol/l for 2-hour PG, 6.1% (43 mmol/mol) for HbA_1c_, 17.0% for GA, and 12.1 μg/mL for 1,5-AG. These results were in accordance with those from the prevalence analysis of DR by decile levels of these measures of glycemia. These findings suggest that the FPG and HbA_1c_ thresholds for diagnosing diabetes in the Japanese population are lower than the current diagnostic criterion, while the 2-hour PG threshold is approximately 11.1 mmol/l, which is comparable to the diagnostic criterion. To our knowledge, the present study is the first report to determine the GA and 1,5-AG thresholds for the diagnosis of diabetes using the prevalence of DR. Furthermore, 2-hour PG had higher sensitivity and larger AUC than other glycemic measures, whereas the AUCs for FPG, HbA_1c_, GA, and 1,5-AG were not significantly different. These findings indicate that 2-hour PG has the highest discriminative ability, and measurements of FPG, HbA_1c_, GA, and 1,5-AG are similar in their ability.

The HbA_1c_ thresholds for identifying presence of DR have varied among prior epidemiological studies of Asian populations, ranging from 6.1% (43 mmol/mol) to 7.0% (53 mmol/mol). In a study in a Singapore population, the optimal HbA_1c_ threshold for DR was 6.6-7.0% (49-53 mmol/mol) [15]. A subanalysis of the DETECT-2, which included three Asian studies in India, Singapore, and Japan, showed that an HbA_1c_ of 6.4% (46 mmol/mol) was the optimal threshold [16]. Similar findings were observed in a Chinese population study (6.4% [46 mmol/mol]) [17]. On the other hand, in the present study, the prevalence of DR increased precipitously when HbA_1c_ levels exceeded 5.9-6.2% (41-44 mmol/mol), and the optimal threshold of HbA_1c_ using the ROC analysis was 6.1% (43 mmol/mol). Importantly, this threshold was identical with that from our previous study in 1998 (NGSP: 6.1% [43 mmol/mol]; JDS: 5.7%) [14]. Furthermore, in another epidemiological study in a Japanese population, the ROC analysis indicated that the highest precision for DR was observed at an HbA_1c_ value of 6.2% (44 mmol/mol) [18]. These findings suggest that the HbA_1c_ threshold in the Japanese population was lower than that of other Asians and also lower than the diagnostic criterion of 6.5% (48 mmol/mol). Although the reason for this difference is unclear, the influence of race and ethnicity on HbA_1c_ levels may contribute to this phenomenon. Some epidemiological studies have shown that South Asians, Hispanics, and Blacks had higher HbA_1c_ levels than non-Hispanic whites, independent of glucose [39,40]. In our subjects, the mean of HbA_1c_ levels (5.5% [37 mmol/mol]) was lower than those in other Asian population studies (6.0-6.5% [42-48 mmol/mol]) [15-17]. Thus, it is speculated that there are racial and ethnic differences in HbA_1c_ levels even among Asians, and this may be the reason for the lower threshold in our subjects. In addition, the aldehyde dehydrogenase 2*2 (ALDH2*2) allele, which is more common in East Asians than in other ethnic groups, has been identified as a genetic risk factor for incident DR in Japanese subjects with diabetes [41], and thus, such a genetic difference in susceptibility to DR might also affect the HbA_1c_ levels associated with incident DR.

The use of HbA_1c_ measurement to diagnose diabetes remains somewhat controversial [42]. Recent epidemiological studies have revealed that HbA_1c_ measurement alone was less sensitive for detecting subjects with diabetes compared to the OGTT [43,44]. However, in our study, the AUCs for HbA_1c_ and FPG were not significantly different. This finding indicates that the discriminative ability of HbA_1c_ for diagnosing diabetes was comparable to that of FPG. Furthermore, HbA_1c_ measurement can be done without fasting or timed samples, and thus it would be suitable for mass screening in general practice. This advantage has implications for the early identification and treatment of undiagnosed diabetes. For these reasons, HbA_1c_ measurement may be an appropriate tool for detecting undiagnosed diabetes. In addition, some clinical and population-based studies, including our previous study, have shown that elevated HbA_1c_ levels were independently associated with cardiovascular disease [45,46], suggesting that HbA_1c_ is also useful as a predictor of macrovascular complications. Therefore, the use of HbA_1c_ to diagnose diabetes will help to prevent both micro- and macrovascular complications of diabetes, which are increasingly recognized as a global health priority.

In the present analysis, although the prevalence of DR was quite small for GA below 16.2-17.5%, it began to rise sharply above these levels, and the optimal threshold of GA using the ROC analysis was 17.0%. Several studies have examined the use of GA levels for detecting diabetes or glucose intolerance, as defined by glucose levels. In a Japanese population study, the ROC analysis for detecting diabetes identified the GA threshold as 15.5% [23], while another study of Japanese subjects reported a GA level of 17.0% as the lower limit of glucose intolerance [21]. A similar threshold of GA was obtained in a Chinese population study (17.1%) [22]. The thresholds in these studies were in good agreement with our findings. Together with those of other studies, our findings suggest that the optimal GA threshold for diagnosing diabetes is likely to be 17.0%.

There have been a few studies evaluating the optimal threshold of 1,5-AG for identifying individuals with diabetes, as defined by a OGTT. A Japanese population study showed that 14.0 μg/mL was the best value for detecting subjects with diabetes [24]. Similar findings were observed among Japanese male workers (14.2 μg/mL) [26]. In a Chinese study, the mean of 1,5-AG levels was 15.0 μg/mL in subjects with newly diagnosed diabetes and 11.8 μg/mL in subjects with known diabetes [25]. However, no study showed an optimal threshold of 1,5-AG using the presence of DR. The present study revealed that the steepest increment in the prevalence of DR occurred when the 1,5-AG levels fell below 9.6-13.5 μg/mL, and that 12.1 μg/mL was the optimal 1,5-AG threshold in the ROC analysis. Further epidemiological studies are needed to verify our findings.

The FPG of 7.0 mmol/l and the 2-hour PG of 11.1 mmol/l for diagnosing diabetes with the current criterion were also derived mainly from studies in Western populations [7]. In our study, the optimal threshold for detecting prevalent DR was 6.5 mmol/l for FPG, and 11.5 mmol/l for 2-hour PG. The 2-hour PG threshold was compatible with that from our previous report (11.1 mmol/l) [14] and another study of a Japanese population (11.0 mmol/l) [29]. Meanwhile, other Asian population studies have reported that the optimal FPG threshold for DR was 7.0 mmol/l in a Japanese population [29], and 7.2 mmol/l in a Chinese population [17]. These findings are inconsistent with ours. However, a recent meta-analysis in Asian and Western populations evaluated the relationship of glucose levels with DR and concluded that the FPG threshold for diagnosing diabetes was 6.5 mmol/l [16]. Furthermore, our prior studies showed that the threshold of FPG for DR was 6.4 mmol/l [14] and that the FPG threshold corresponding to a 2-h PG of 11.1 mmol/l was 6.2 mmol/l [47]. These results were very similar to those of the present study. Taken together, these findings imply that, in a Japanese population, the threshold of FPG for diabetes is lower than the diagnostic criterion of 7.0 mmol/l, while the threshold of 2-hour PG is 11.1 mmol/l, which is in accord with the diagnostic criterion.

The present study showed that among the five glycemic measures, 2-hour PG had not only the highest sensitivity but also the largest AUC to identify the presence of DR. These results suggest that the performance and discriminative ability of 2-hour PG for diagnosing diabetes were superior to those of other glycemic measures. Oxidative stress is known to be one of the crucial contributors in the pathogenesis of DR [48]. It has also been reported that acute hyperglycemia had a more specific triggering effect on oxidative stress than chronic sustained hyperglycemia [49,50]. Thus, 2-hour PG values can be considered a better marker of oxidative stress levels arising from acute hyperglycemia than FPG, HbA_1c_, GA, and 1,5-AG values. Furthermore, a prospective study demonstrated that postprandial plasma glucose was a stronger predictor of the progression of DR than HbA_1c_ in Japanese subjects with diabetes [51]. Taken together, these findings imply that 2-hour PG levels may be more strongly associated with DR than other glycemic measures. This may explain why 2-hour PG has a high diagnostic accuracy for DR. On the other hand, the AUCs for GA and 1,5-AG did not significantly differ from those for FPG and HbA_1c_, suggesting that GA and 1,5-AG are acceptable alternatives for the diagnosis of diabetes, and these two measures may be particularly useful for individuals with anemia, renal disease or hemoglobinopathy, for whom interpretation of HbA_1c_ values is problematic. However, the 1,5-AG levels had smaller AUC with lower sensitivity than other glycemic measures. One possible explanation for this phenomenon may be that 1,5-AG levels reflect the degree of glycosuria rather than glucose levels [28], while other glycemic measures directly indicate the degree of hyperglycemia. In addition, it has been reported that 1,5-AG levels were influenced by individual difference in their renal thresholds for glucose [52]. These facts might be the reason for the relatively low discriminative ability of 1,5-AG in our study.

The strengths of our study include the population-based design, high participation rate, and availability of data to evaluate the five glycemic measures. In addition, it is noteworthy that the FPG, 2-hour PG, and HbA_1c_ thresholds in the present study were nearly the same as those from our previous study [14], suggesting the high reproducibility of the results in our population. However, some limitations should also be mentioned. First, our analyses included subjects with antidiabetic medications. Hypoglycemic medications could have affected the levels of glycemia. The optimal thresholds remained substantially unchanged, except for GA, after excluding subjects who received hypoglycemic medications (FPG: 6.3 mmol/l; 2-hour PG: 11.5 mmol/l; HbA_1c_: 6.2% [44 mmol/mol]; GA: 20.5%; and 1,5-AG: 12.1 μg/mL). However, the precision of this finding may be limited, because of the small number of cases of DR among those not taking hypoglycemic medications. Second, the values of HbA_1c_ were not measured by high-performance liquid chromatography (HPLC) as used in the Diabetes Control and Complications Trial, although the method and reagent used to measure HbA_1c_ in this study have since been NGSP-certified. It would be preferable to measure HbA_1c_ by HPLC to make the results of our study more comparable to those of other studies. Third, the GA and 1,5-AG levels were measured in serum conserved at -80°C for 5 years. However, the stability of GA and 1,5-AG measurements in frozen stored serum sample was preserved [53,54]. Fourth, this study is a cross-sectional design, which might have affected the threshold values of glycemic measures. Diagnostic thresholds would ideally be derived from prospective studies that examine the relationship between measures of glycemia and incident microvascular complications. Lastly, the influence of factors that may affect HbA_1c_ levels, such as anemia, renal failure, and hemoglobinopathy should be considered. We performed sensitivity analyses excluding subjects with anemia or renal failure, and the optimal threshold of HbA_1c_ remained unchanged (6.1% [43 mmol/mol]). Furthermore, the prevalence of hemoglobinopathy in Japan was reported to be very low (0.04%) [55]. Therefore, the influence of this limitation would have been small.

## Conclusions

The present analysis showed that, in a Japanese population, the FPG and HbA_1c_ threshold for diagnosing diabetes was lower than the current diagnostic criterion, while the 2-hour PG threshold was consistent with the diagnostic criterion, and the discriminative ability of 2-hour PG was superior to other glycemic measures. These findings suggest that the threshold of 2-hour PG is 11.1 mmol/l, regardless of race, whereas ethnic-specific thresholds of FPG and HbA_1c_ may be necessary. This study also demonstrated the potential applicability of GA and 1,5-AG measurements as a diagnostic tool for diabetes. Further prospective studies are needed to verify these findings, and investigations of HbA_1c_ levels in the intermediate range are also required.

## Abbreviations

HbA1c: Hemoglobin A_1c_; DR: Diabetic retinopathy; FPG: Fasting plasma glucose; GA: Glycated albumin; 1,5-AG: 1,5-anhydroglucitol; PG: Postload glucose; OGTT: Oral glucose tolerance test; Hb: Hemoglobin; NGSP: National Glycohemoglobin Standardization Program; JDS: Japan Diabetes Society; eGFR: Estimated glomerular filtration rate; BMI: Body mass index; ETDRS: Early Treatment of Diabetic Retinopathy Study; ROC: Receiver operating characteristic; AUC: Area under the receiver operating characteristic curve; CI: Confidence interval; HPLC: High-performance liquid chromatography.

## Competing interests

The authors declare that they have no competing interests.

## Authors’ contributions

NM contributed to the study concept and design, data collection, data analysis, data interpretation, and drafting of the manuscript. MY contributed to the data collection, data interpretation, and drafting of the manuscript. TN, JH, YH, FI, and MF contributed to the data collection and data interpretation. TH and DK measured the samples and contributed to the data interpretation. MK, UN, and TK contributed to the data interpretation and the critical revision of the manuscript for important intellectual content. YK contributed to the data collection, data interpretation, drafting of the manuscript, obtained funding, and study supervision. All authors provided critical review of the draft and approved the final version.
